# Disseminated Multi-system Sarcoidosis Mimicking Metastases on ^18^F-FDG PET/CT

**DOI:** 10.4274/mirt.29200

**Published:** 2018-06-07

**Authors:** William Makis, Mark Palayew, Christopher Rush, Stephan Probst

**Affiliations:** 1 Cross Cancer Institute, Department of Diagnostic Imaging, Edmonton, Canada; 2 Jewish General Hospital, Department of Nuclear Medicine, Montreal, Canada

**Keywords:** Sarcoidosis, artifact, mimic, lymphadenopathy, metastases, 18F-fluorodeoxyglucose, positron emission tomography

## Abstract

A 60-year-old female with no significant medical history presented with hematuria. A computed tomography (CT) scan revealed extensive lymphadenopathy with hypodensities in the liver and spleen, and she was referred for an ^18^F-fluorodeoxyglucose (^18^F-FDG) positron emission tomography/CT (PET/CT) study to assess for malignancy of unknown primary. PET/CT revealed extensive ^18^F-FDG avid lymphadenopathy as well as innumerable intensely ^18^F-FDG avid lung, liver and splenic nodules, highly concerning for malignancy. A PET-guided bone marrow biopsy of the posterior superior iliac spine revealed several non-necrotizing, well-formed granulomas, consistent with sarcoidosis. The patient was managed conservatively and remained clinically well over the subsequent 9 years of follow-up.

## Abstract (tr)

Tıbbi anamnezinde özellik olmayan 60 yaşında bir kadın hematüri ile başvurdu. Bilgisayarlı tomografide (BT) yaygın lenfadenopatiyle birlikte karaciğer ve dalakta hipodens alanlar saptanması üzerine primeri bilinmeyen malignite değerlendirilmesi amacıyla ^18^F-fluorodeoksiglukoz (^18^F-FDG) pozitron emisyon tomografisi/BT (PET/BT) için yönlendirildi. PET/BT’de ^18^F-FDG tutan yaygın lenfadenopatiler ve ölçülemeyecek kadar fazla yoğun ^18^F-FDG tutan akciğer, karaciğer ve dalak nodülleri saptandı, malignite açısından şüpheli bulundu. PET-kılavuzluğunda posterior superior spina iliacadan yapılan kemik iliği biyopsisinde sarkoidoz ile uyumlu non-kazeifiye granülomlar saptandı. Hasta konservatif olarak takip edildi ve 9 yıllık takip süresinde klinik sorun oluşmadı.

## Figures and Tables

**Figure 1:**
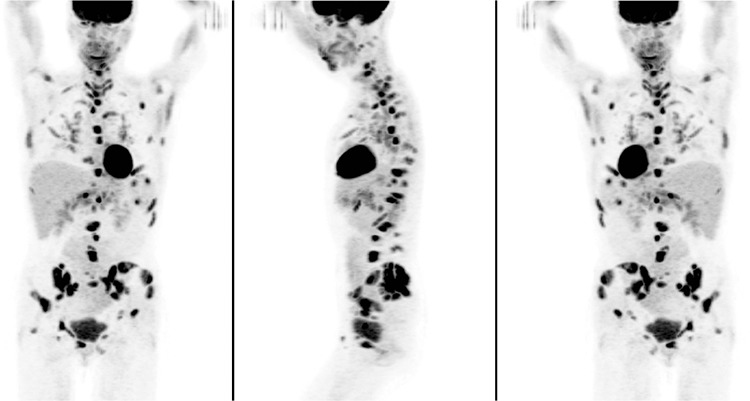
A 60-year-old woman, non-smoker with no significant medical history presented with recurrent hematuria. Computed tomography (CT) abdomen/pelvis identified intra-abdominal, retroperitoneal and inguinal lymphadenopathy, and small hepatic/splenic hypodensities, prompting referral for positron emission tomography/CT (PET/CT) to assess for malignancy of unknown origin. Maximum intensity projection images revealed widespread foci of intense ^18^F-FDG uptake throughout the skeleton and soft-tissues.

**Figure 2:**
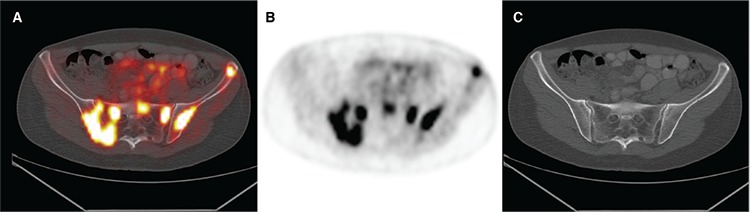
Transaxial (A) PET/CT fusion, (B) PET, and (C) CT images revealed extensive bone involvement of the skull base, right clavicle, spine, multiple ribs, sternum, proximal left humerus, right femur and extensively throughout the pelvis with maximum standardized uptake value (SUV_max_) 8.3.

**Figure 3:**
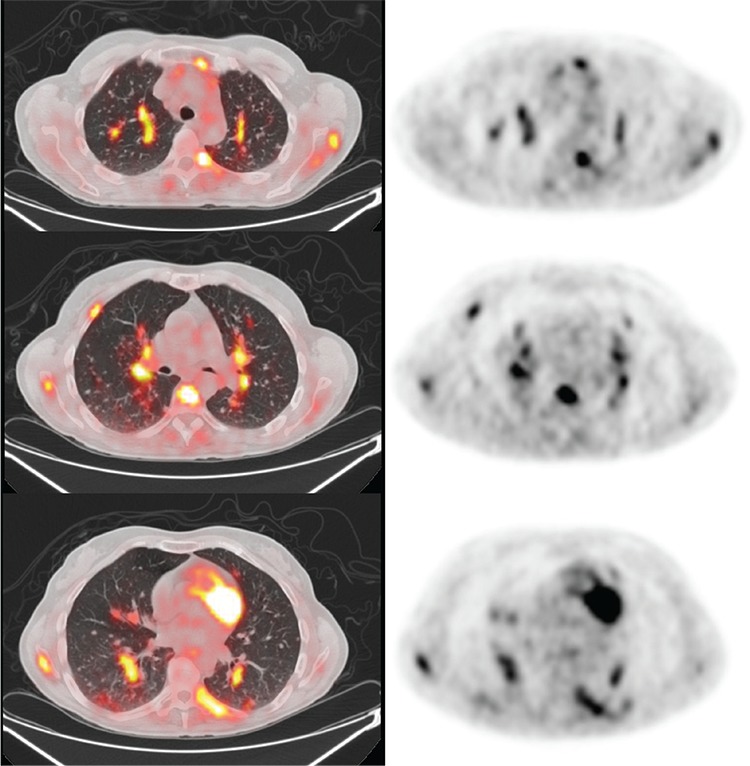
Intense ^18^F-FDG uptake was also noted in numerous peribronchovascular and subpleural nodules in both lungs (largest was 1.0 cm in diameter with SUV_max_ 3.2).

**Figure 4:**
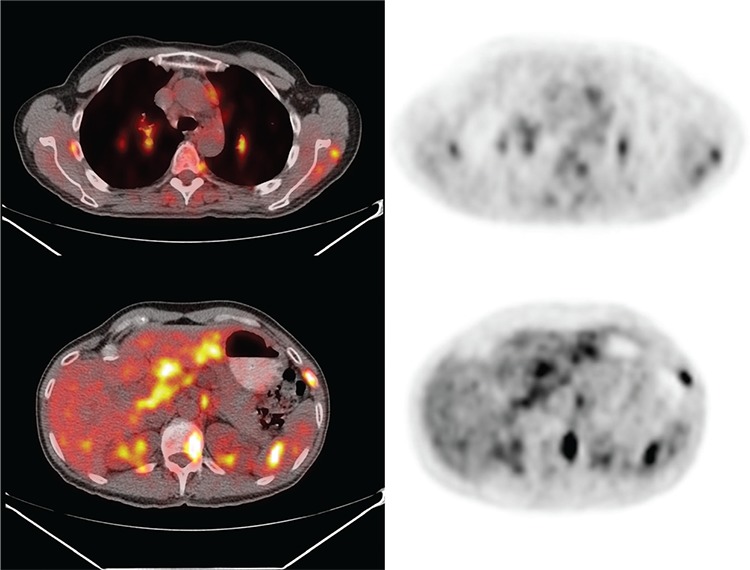
There was widespread adenopathy including left retromandibular, mediastinal, hilar, abdominal, retroperitoneal and inguinal nodes (largest mediastinal node measured 1.4 cm, and the most metabolically active was a right perihilar lymph node with SUV_max_ 5.2). The innumerable liver and spleen hypodensities identified on CT were intensely ^18^F-FDG avid with SUV_max_ 4.3 in the liver and 5.9 in the spleen. No primary malignancy was identified, but findings were interpreted as highly concerning for disseminated metastatic disease. The patient remained asymptomatic. PET-guided bone marrow biopsy of the left posterior superior iliac spine revealed several non-necrotizing, well-formed granulomas. These granulomas were paratrabecular in distribution and were composed of tightly apposed epithelioid histiocytes, with occasional multinucleated giant cells, consistent with sarcoidosis.

**Figure 5:**
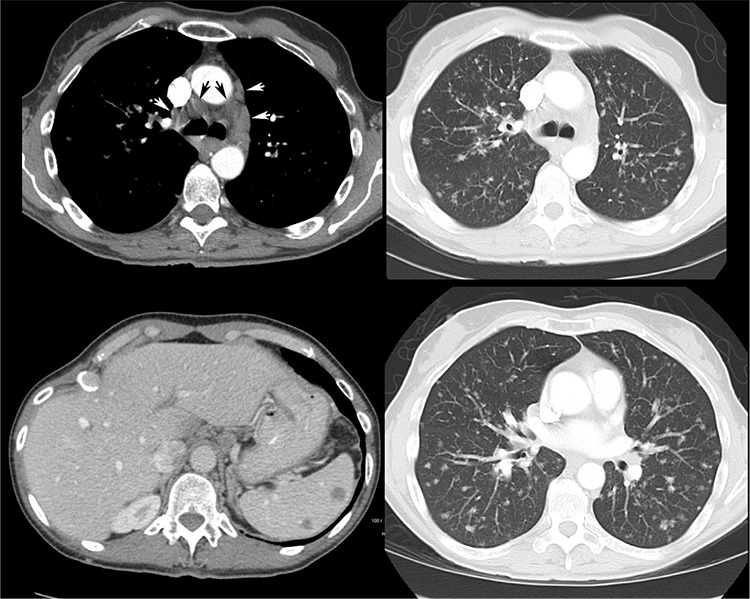
Follow-up diagnostic CT of chest/abdomen/pelvis performed 6 months later, again revealed extensive poorly marginated lung nodules, thoracic and abdominal lymphadenopathy, and splenic and hepatic hypodensities, unchanged as compared to prior PET/CT and consistent with stable granulomatous disease. The patient was managed conservatively with observation and remained malignancy-free over the subsequent 9 years of clinical and imaging follow-up. Sarcoidosis is a chronic multisystem granulomatous disorder of unknown etiology, which is characterized pathologically by non-caseating granulomas that can present almost anywhere in the body (1,2,3,4). ^18^F-FDG uptake in the granulomas of sarcoidosis can be very intense, likely due to metabolic activity of activated macrophages (2,3,5). ^18^F-FDG-avid lesions of skeletal sarcoidosis cannot be reliably differentiated from metastases or other benign bone processes (Paget’s disease, fibrous dysplasia, giant cell tumors, osteomyelitis) on the basis of semi-quantitative (SUV), visual or other analysis, and therefore remain a pitfall of oncologic PET/CT interpretation (6,7,8,9,10). ^18^F-FDG uptake in pulmonary sarcoidosis lesions has been described and the severity of pulmonary involvement has been shown to be associated with ^18^F-FDG activity in persistently symptomatic sarcoidosis patients (11). CT studies show presence of hepatic and splenic nodules in approximately 5-15% of sarcoidosis patients and splenic nodules tend to be larger than hepatic nodules (12). Intense ^18^F-FDG uptake in hepatic and splenic sarcoidosis lesions has been previously described (13). This case shows an uncommon presentation of disseminated sarcoidosis in the skeleton, lymph nodes, as well as organs such as the lungs, liver and spleen. This impressive pattern of disseminated ^18^F-FDG uptake can be easily mistaken for extensive metastatic disease when interpreting oncologic PET/CT studies.
